# Systemic and intrathecal baclofen produce bladder antinociception in rats

**DOI:** 10.1186/s12894-021-00899-0

**Published:** 2021-10-04

**Authors:** Timothy J. Ness, Alan Randich, Xin Su, Cary DeWitte, Keith Hildebrand

**Affiliations:** 1 Department of Anesthesiology and Perioperative Medicine, University of Alabama at Birmingham, BMR2-208, 901 19th Street South, Birmingham, AL 35294 USA; 2 Medtronics, Inc., Minneapolis, MN USA

**Keywords:** Interstitial cystitis/bladder pain syndrome, Antinociception, Urinary bladder, GABA_B_ receptors

## Abstract

**Background:**

Baclofen, a clinically available GABA_B_ receptor agonist, produces non-opioid analgesia in multiple models of pain but has not been tested for effects on bladder nociception.

**Methods:**

A series of experiments examined the effects of systemic and spinally administered baclofen on bladder nociception in female anesthetized rats. Models of bladder nociception included those which employed neonatal and adult bladder inflammation to produce bladder hypersensitivity.

**Results:**

Cumulative intraperitoneal dosing (1–8 mg/kg IP) and cumulative intrathecal dosing (10–160 ng IT) of baclofen led to dose-dependent inhibition of visceromotor responses (VMRs) to urinary bladder distension (UBD) in all tested models. There were no differences in the magnitude of the analgesic effects of baclofen as a function of inflammation versus no inflammation treatments. Hemodynamic (pressor) responses to UBD were similarly inhibited by IT baclofen as well as UBD-evoked excitatory responses of spinal dorsal horn neurons. The GABA_B_ receptor antagonist, CGP 35,348, antagonized the antinociceptive effects of IT baclofen on VMRs in all tested models but did not affect the magnitude of the VMRs by itself suggesting no tonic GABA_B_ activity was present in this preparation. Tolerance to a seven day continuous IT infusion of baclofen was not observed.

**Conclusions:**

These data provide support for a clinical trial of baclofen as a non-opioid treatment of human bladder pain.

## Background

Baclofen has been clinically available for over 60 years [1]. It is a gamma amino butyric acid (GABA) derivative that is thought to work via the metabotropic GABA_B_ receptor. Used mainly for the treatment of muscle spasticity, it has also found a role in the treatment of neuropathic pain [2–4]. Recent experiments reported by ourselves [5] which assessed the effects of commonly employed adjuvant agents on peripheral nerve stimulation-related neuromodulation identified that baclofen may have an analgesic effect on bladder nociception. However, the rats used in those particular experiments had extensive surgeries and received numerous other manipulations. Therefore, to properly assess the effects of baclofen on responses to urinary bladder distension (UBD), experiments need to be performed in isolation from those other manipulations. Moreover, concerns about opioid overdose/misuse have become of paramount clinical concern [6] and so it is important to determine whether non-opioid drugs which are already approved for use in humans may be repurposed for the treatment of specific types of pain such as bladder pain.

Numerous basic science reports suggest that baclofen, administered either systemically or intrathecally, inhibits nociception. It is known to produce analgesia by itself as measured in rats and mice in the hot plate, tail flick and acetic acid-induced writhing [7–21]. Baclofen also produces analgesia in primates in the formalin test [22]. In chemotherapy-induced neuropathic pain models baclofen produces significant but erratic analgesia [4]. It has also been demonstrated to have peripheral analgesic effects in a mouse arthritis model [23]. In humans, baclofen has had a particular role as an analgesic for cranial nerve-related neuropathic pains such as trigeminal neuralgia [2]. Anecdotal reports (e.g. [3]) suggest it also works for postherpetic neuralgia in facial distributions. In humans, the spinal administration of baclofen has also demonstrated short-term analgesic effects on spinal cord injury-related pain and post-stroke pain [24] and provides analgesia following surgery, in most cases by augmenting opioid effects [25–27].

Despite these encouraging basic science and clinical reports, baclofen has not been used frequently for non-neuropathic pain in humans. This is unfortunate because baclofen may have other beneficial effects related to the attenuation of abuse/misuse of opioids. In rat models, baclofen dose-dependently reduced heroin-seeking behavior [28, 29], reversed behavioral sensitization to morphine [30], prevented reinstatement of heroin-seeking behaviors [28] and enhanced extinction of opiate and methamphetamine-induced conditioned place preference [31, 32]. These effects of baclofen on reward and dependence systems are particularly important given the present societal concerns related to opioid overuse and misuse. Having an effective non-opioid therapy (or supplemental therapy), such as baclofen, as an alternative to high doses of opioids is therefore both desirable and an ethical imperative. The following studies were therefore performed to test the potential utility of baclofen as an analgesic agent in the treatment of bladder pain and more generally, to enhance the database for alternatives to the use of opioids in the treatment of chronic pain.

## Experimental procedures

### Overview

In the present experiments, we examined the antinociceptive effects of both systemic and spinal administration of baclofen. We used several models of bladder nociception with endpoints which included spinal dorsal horn neuronal responses, UBD-evoked changes in arterial blood pressure and the VMR (abdominal contractions) evoked by UBD. In addition to normal, healthy rats, we also assessed responses in rats whose bladders were made hypersensitive to bladder distension by inflammation. These studies were all approved by the UAB Institutional Animal Care and Utilization Committee.

### Animal subjects

A total of 205 Sprague–Dawley female rats obtained from Harlan/Envigo (Prattville, AL) were used as adults (mean weight 283 ± 12 g) in all experiments. In some experiments, rats were raised from birth, treated as pups and allowed to mature to adulthood (description in section “Neonatal bladder inflammation (NBI)”). All rats were housed with food and water available on an ad libitum basis. A 12:12-h light:dark cycle was maintained, where lights were off between 6:00 pm and 6:00 am. Female rats were exclusively employed for practical reasons (difficult to cannulate male rats’ urethras) but can be justified by the observation that VMRs and cardiovascular responses to urinary bladder distension in female rats are more reliable and robust than in male rats [33] and chronic bladder pains have a high female prevalence in humans [34]. There was no attempt to control for estrous cycle, as the focus of this study was not on estrous-related changes in pain, and we have previously shown that hormone fluctuations due to the estrous cycle do not alter the vigor of UBD-evoked VMRs in rats without bladder inflammation when the current methodology is employed [35].

### Adult bladder inflammation (ABI)

As adults, some rats received a treatment 24 h prior to UBD testing on the following day. At that time, animals were anesthetized with inhaled isoflurane and oxygen (5% for induction, 2% for maintenance) and separated into groups receiving either no treatment or intravesical zymosan. Intravesical zymosan treatment has been demonstrated to produce a robust bladder inflammation and hypersensitivity to UBD [36]. Zymosan-treated animals had their urinary bladders catheterized with a 22-gauge angiocatheter via the urethra. Zymosan (0.5 ml, 1% in saline) was administered intravesically for 30 min and drained. Rats in the *anesthesia only* control group were maintained on 2% isoflurane for 30 min, immediately after induction with 5% isoflurane. All animals received ampicillin at the end of the procedure (50–100 mg/kg, s.c.).

### Neonatal bladder inflammation (NBI)

Some groups of female rat pups were given three daily neonatal treatments of zymosan beginning on post-natal day 14 (P14–16). Each rat was anesthetized with isoflurane (5% induction; 2% maintenance) delivered by mask. In one group, a 1% zymosan (0.1 ml) solution was instilled intravesically via a 24 gauge angiocatheter placed through the urethra and allowed to dwell for 30 min. The bladder then was drained, the catheter removed, and the rat permitted to recover. The control group only received anesthesia and no catheterization or zymosan treatment. All rats were kept on a warmed heating pad during treatments and received ampicillin (0.05 mg in 0.05 ml s.c.) at the end of each treatment before being returned to their dams. All rats were weaned at 3 weeks and then raised to adulthood before use (12–15 weeks of age). This treatment has been demonstrated to produce hypersensitivity to UBD and is described more completely elsewhere [37].

### Reflex responses to urinary bladder distension (UBD)

Animals were anesthetized with urethane (1.2 g/kg s.c.) and/or isoflurane (2–5% during surgery, later reduced to < 0.75%). A 22-gauge polytetrafluoroethylene angiocatheter (Johnson and Johnson, Arlington TX) was placed into the bladder via the urethra and held in place by a tight suture around the distal urethral orifice. Silver wire electrodes were inserted into the external oblique musculature immediately superior to the inguinal ligament for recording of abdominal EMG activity. In some cases, a carotid arterial catheter was placed to allow for hemodynamic measures. Following surgery, anesthesia was reduced until flexion reflexes were present in the hind limbs but spontaneous escape behaviors were absent. In most experiments, the primary response measure was the visceromotor reflex (VMR), an abdominal contraction evoked by UBD which consisted of air distension of the urinary bladder using the intravesical catheter. An in-line, pneumatically-linked, low volume pressure transducer was used to monitor distending pressures. VMRs, recorded as electromyographic activity of the abdominal musculature, was measured via the electrodes using standard differential amplification and rectification and saved on a computer (Spike 2 software, Cambridge Electronic Design, UK). “Evoked” VMR responses were defined as the mean rectified electromyographic activity (in mV) during 20 s of UBD minus the mean baseline electromyographic activity (in mV) measured in the period immediately preceding the onset of UBD. Hemodynamic responses to UBD were defined as the maximal sustained change in mean arterial pressure measured during UBD minus the mean arterial pressure measured immediately prior to UBD. This model system has been described more extensively elsewhere [38, 39].

### Intrathecal catheters

In experiments in which selective spinal action of drugs was studied a 7.8 cm catheter made of PE10 tubing was inserted via an incision in the atlanto-occipital membrane following surgical exposure and threaded down through the subarachnoid space to the lumbosacral region under deep isoflurane/oxygen anesthesia. In most cases, the catheter was used immediately in non-survival experiments (as per sections “Protocol for spinal baclofen cumulative dosing experiments” and “Protocol for assessing effects on hemodynamic responses to UBD”), but in 13 rats the catheter was attached to an Alzet 2001 osmotic minipump (Durect Corp, Cupertino, CA, USA) allowing for a 7-day infusion of either normal saline or a baclofen solution as described in section “Dorsal horn spinal neuronal responses to UBD”.

### Protocol for systemic baclofen cumulative dosing experiments

Repeated 60 mmHg UBDs were administered with a 3 min inter-trial interval until stable VMRs were established. Graded, constant-pressure air distensions of the urinary bladder (20 s duration; 3 min inter-trial interval) of ascending pressures at intervals of 10, 20, 30, 40, 50 and 60 mmHg were then administered to quantify the graded stimulus–response. A cumulative dosing paradigm was then initiated with consecutive doses of 1, 1, 2 and 4 mg/kg of baclofen (Sigma-Aldrich, St. Louis, MO, USA; Cat. No. B5399) or equal volumes of normal saline (1 ml/kg) administered IP for cumulative doses of 1, 2, 4 and 8 mg/kg respectively in the case of baclofen. The saline injections served as a repeated measures control procedure. Fifteen minutes following each injection graded stimulus–response measures (10–60 mm Hg, 20 s UBDs) were re-determined using the method described above.

### Protocol for spinal baclofen cumulative dosing experiments

Rats were prepared following a surgical procedure similar to that used in section “Protocol for systemic baclofen cumulative dosing experiments”, but with the addition of an acutely placed IT catheter. A protocol similar to that described in section “Protocol for systemic baclofen cumulative dosing experiments” was then performed using consecutive doses of 10, 10, 20, 40 and 80 ng of baclofen (or equal volumes of normal saline) dissolved in 10 µl of normal saline followed by a 10 µl normal saline flush through the IT catheter. Graded stimulus–response measures were determined 15 min following each baclofen/saline injection.

### Protocol for assessing effects on hemodynamic responses to UBD

In these rats, a single 40 ng dose of IT baclofen (10 µl injection; 10 µl normal saline flush) or equal volume of IT normal saline was administered and responses to repeated UBDs (60 mm Hg, 20 s, 3 min intervals) measures, allowing for assessment of the time course of the baclofen effect. In this case, responses included both VMRs and hemodynamic responses measured using a carotid arterial catheter. The IT saline-treated rats served as a control for repeated measures.

### Protocol for assessing actions of baclofen via GABA-B receptors

To verify that baclofen was acting through GABA_B_ receptor mechanisms, an antagonist was administered IT prior to administration of baclofen. Specifically, these rats received repeated UBDs (60 mm Hg, 20 s 3 min ITI). After VMRs to UBD were stable (± 20%), responses to graded UBD (10–60 mm Hg, 20 s) were obtained. Rats then received a single IT injection of either CGP35348 (30 µg; Tocris Biosciences, Minneapolis, MN, USA; Cat. No. 1245) or an equal volume of normal saline (10 µl) followed by a 10 µl normal saline flush. Fifteen minutes after the injection repeat graded UBD stimulus–response measures were obtained. Rats then received an IT injection of baclofen (40 ng) in normal saline or an equal volume of normal saline (10 µl) followed by a 10 µl normal saline flush. Fifteen minutes after this injection, repeat graded UBD stimulus–response measures were obtained. These dosing combinations resulted in three separate measures in four separate groups.

### Protocol for assessing tolerance to spinal baclofen

Thirteen rats which had received neonatal bladder inflammation (section “Reflex responses to urinary bladder distension (UBD)”) had IT catheters placed (section “Intrathecal catheters”) and attached to seven day minipumps that were then secured in the subcutaneous tissues between the rats’ scapulae. Incisions were closed with sutures and the rats allowed to recover. Half of the rats received an infusion of baclofen at a rate of 1 µl/h or 20 ng/h for 7 days. The other half of the rats received a normal saline infusion at the same rate. The day before testing, all rats were re-anesthetized with isoflurane, adult bladder inflammation induced (section “Adult bladder inflammation (ABI)”) and their IT catheters externalized and minipumps removed. They were allowed to recover overnight and then underwent cumulative dosing of IT baclofen in a fashion identical to that noted in section “Protocol for spinal baclofen cumulative dosing experiments”.

### Dorsal horn spinal neuronal responses to UBD

Animals were anesthetized *w*ith isoflurane (5%) and a tracheal cannula placed allowing for mechanical ventilation. The cervical spinal cord was exposed surgically, injected with 50 µl of 1% lidocaine solution and subsequently transected using a sharp scalpel. The brain was then pithed mechanically, anesthesia discontinued and the rats allowed to recover until demonstrating robust hindlimb flexion reflexes in response to paw pinching (typically 1–2 h). A laminectomy was then performed and the dura incised exposing the L6-S2 spinal segments since these are known sites for spinal processing of afferent information from the bladder [40]. The vertebral column was clamped both rostrally and caudally to the laminectomy for stabilization. Skin flaps were arranged to form a protective coating for exposed tissue except for the site of recording which was covered with warmed mineral oil. A PE10 catheter was secured at the edge of the exposed spinal dorsal horn for future use in the topical administration of drugs. Tungsten microelectrodes (MicroProbe, Clarksburg, MD; 1.2–1.8 MOhm) were used for conventional extracellular single-unit recording. The dorsal horn 0–1.0 mm from midline and 0–1.2 mm below cord dorsum was searched using microelectrodes positioned using a stereotaxic apparatus. All units responded in a consistent excitatory fashion to UBD. To quantify neuronal responses, units were displayed oscillographically for continuous monitoring, discriminated conventionally from background, converted into uniform pulses and counted and saved by computer. The total number of unit action potentials (discharges) were counted in 10 s epochs before, during and after the UBD stimulus. Evoked Activity of the dorsal horn neurons was defined as the number of unit discharges during UBD minus the level of activity immediately preceding the onset of UBD. Because responses of different neurons to the same distending stimulus naturally vary in maximal response and total number of unit discharges, each unit’s response was normalized to that produced by the 60 mm Hg response for purposes of within- and between-group comparisons. Noxious and non-noxious somatic stimuli were also presented to each neuron and excitatory/inhibitory responses determined in a fashion similar to that which we have previously published [39]. Following characterization, the effect of the spinal application of 160 ng baclofen or an equal volume of normal saline (20 µl) on spinal dorsal horn neuronal responses to repeated UBD (60 mm Hg, 20 s; 3 min intervals) was determined by measuring multiple responses prior to and following drug administration. All of the rats studied in this protocol had also experienced neonatal bladder inflammation and adult bladder inflammation as per sections “Adult bladder inflammation (ABI)” and “Reflex responses to urinary bladder distension (UBD)”.

### Statistical analyses

Statistics are presented as the mean ± S.E.M in the graphs. Area-Under-the-Curve (AUC) statistics were generated as measures of global responses to graded UBD and reported as a percentage of mean pre-drug measures. In some studies, repeated measures ANOVAs were performed followed by post-hoc analyses of means when appropriate. Paired t-tests of pre/post measures were used for comparisons when appropriate.

## Results

### IP baclofen produced dose-dependent inhibition of VMRs to UBD

The upper portion of Table 1 presents mean Area-Under-the-Curve (AUC) measures normalized as a percentage of the pre-drug measures. These values were obtained following cumulative IP dosing of baclofen (1, 2, 4, and 8 mg/kg) or repeated doses of saline in otherwise healthy adult rats which had not experienced any neonatal bladder inflammation (NBI) or adult bladder inflammation (ABI) and so designated the “No NBI, No ABI Groups” and in adult groups which had received both NBI and ABI pretreatments, the “NBI—ABI Groups.”. Notably, the predrug VMRs were more robust in the NBI-ABI Groups compared with the No NBI-No ABI Groups: predrug AUCs were 115 ± 14 V*mmHg versus 78+6 V*mmHg respectively (p = 0.0188 for unpaired t-test, n = 16/group). Baclofen dosing, as compared with repeated saline dosing, produced statistically significant, dose-dependent suppression of the normalized AUC measures. This suppression was similar in both pretreatment groups.

**Table 1:** Dose-dependent inhibitory effects resulting from cumulative doses of baclofen versus repeated doses of saline on visceromotor responses to urinary bladder distension measured as % baseline AUC

	Pretreaments				
	NBI	No NBI		NBI	No NBI
	Plus ABI	No ABI		Plus ABI	No ABI
*Cumulative dose IP*	n = 8	n = 8	*Repeated dose IP*	n = 8	n = 8
Baclofen 1 mg/kg	115.2 ± 23.4	113.0 ± 15.2	Saline 1^st^	142.5 ± 16.8	154.6 ± 9.1
Baclofen 2 mg/kg	101.5 ± 25.4	96.0 ± 17.8*	Saline 2^nd^	145.9 ± 19.5	156.6 ± 8.4
Baclofen 4 mg/kg	57.5 ± 22.2*	67.9 ± 15.3*	Saline 3^rd^	145.5 ± 18.0	142.9 ± 11.2
Baclofen 8 mg/kg	17.2 ± 6.8*	21.0 ± 9.5*	Saline 4^th^	156.7 ± 19.8	141.8 ± 8.8
*Cumulative dose IT*	n = 8	n = 8	*Repeated dose IT*	n = 6	n = 6
Baclofen 10 ng	104.6 ± 5.3	119.4 ± 16.4	Saline 1^st^	110.8 ± 9.3	111.2 ± 10.2
Baclofen 20 ng	82.0 ± 7.2	89.2 ± 12.0	Saline 2^nd^	88.3 ± 18.2	94.9 ± 9.9
Baclofen 40 ng	49.9 ± 11.8*	64.7 ± 15.8*	Saline 3^rd^	114.2 + 24.2	112.0 ± 14.9
Baclofen 80 ng	24.5 ± 8.3*	30.5 ± 15.1*	Saline 4^th^	114.0 ± 25.1	121.1 ± 15.9
Baclofen 160 ng	9.9 ± 4.6*	9.1 ± 9.1*	Saline 5^th^	93.7 ± 21.1	142.0 ± 22.7

Data represents mean ± SEM

AUC indicates Area-Under-the-Curve, a statistic describing vigor of visceromotor responses to graded urinary Bladder distension (10–60 mm Hg, 20 s). See text for more complete description

*NBI* neonatal bladder inflammation—pretreatment as defined in section “Neonatal bladder inflammation (NBI)” of text; *ABI* adult bladder inflammation—pretreatment as defined in section “Adult bladder inflammation (ABI)” of text, *mcg* microgram, *ng* nanogram, *kg* kilogram, *IP* intraperitoneal, *IT* intrathecal

*Statistically significant difference from Saline-dosed rats in same Pretreatment category (*p* < 0.05, unpaired t-test)

### IT baclofen produced dose-dependent inhibition of VMRs to UBD

**Fig. 1:**
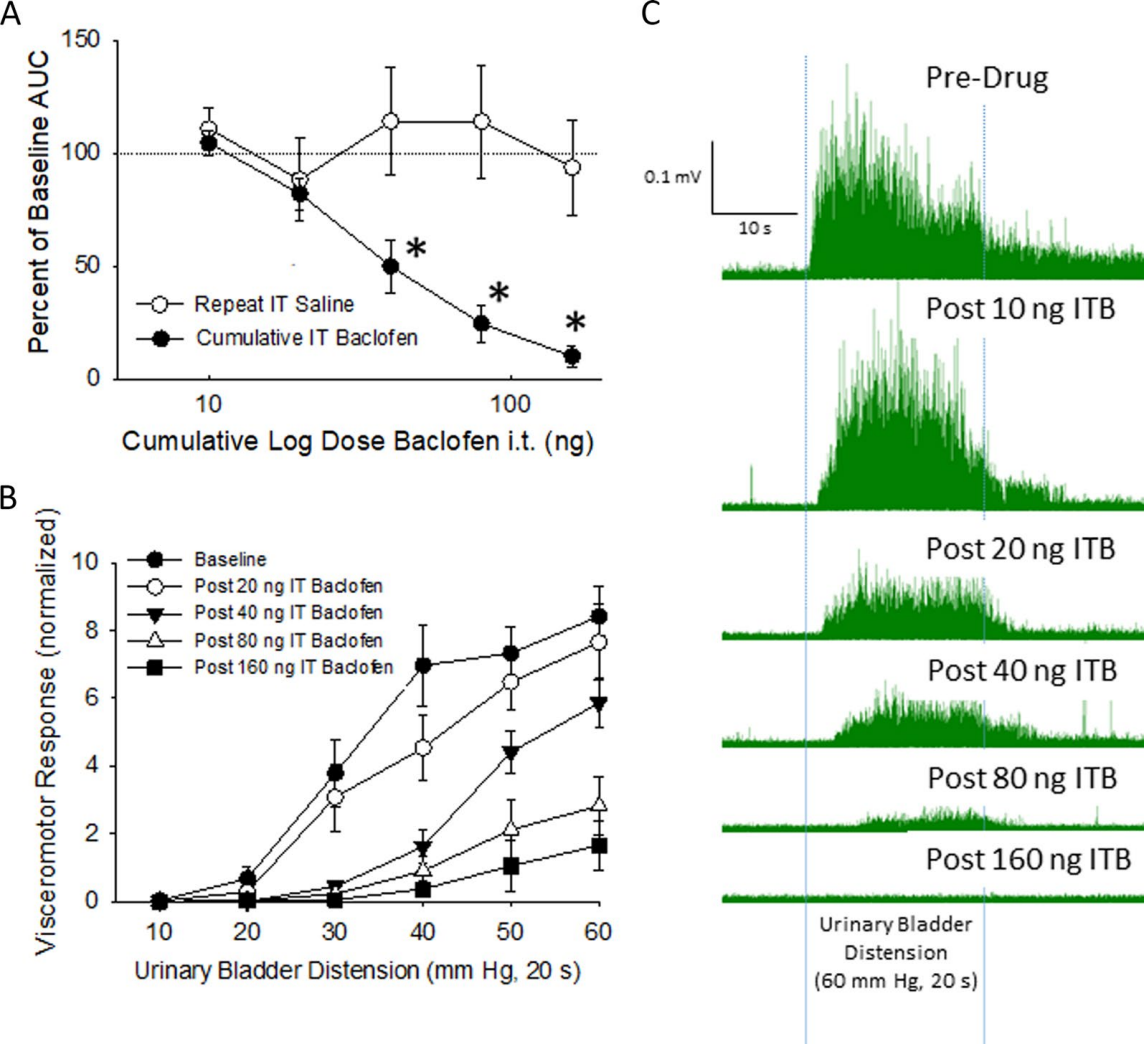
Cumulative dosing of IT baclofen. **a** Abdominal EMG responses to graded UBD (10–60 mmHg) were quantified as a mean Area-Under-the-Curve (AUC) for each dose of baclofen tested (n = 8; cumulative doses values of 0, 10, 20, 40, 80 and 160 ng) and presented on a log scale. These responses were significantly different for AUC measures in IT saline-treated rats (n = 8). * indicates p < 0.05 for post hoc comparisons. **b** Stimulus–response functions for the IT baclofen used to generate the AUC data in panel A and Table 1. **c** Individual example of rectified electromyographic activity in a single rat before and after stated doses of IT baclofen

Figure 1 and the lower portion of Table 1 present the results of the cumulative IT dosing of baclofen (10, 20, 40, 80 and 160 ng) or repeated doses of normal saline. There were no significant differences between-groups in baseline AUC measures prior to dosing with baclofen (NBI-ABI predrup AUC 184+28 V*mmHg versus the No NBI-NoABI predrug AUC of 128+43 V*mmHg (n = 12/group) although this lack of difference may have represented a smaller sample size and greater variability of VMRs in rats which had IT catheters placed. Similar to the IP-treated rats described in section “IP baclofen produced dose-dependent inhibition of VMRs to UBD”, IT baclofen-treated groups, when compared with their corresponding IT saline-treated groups manifested significant dose-dependent suppression of their VMRs as quantified by the AUC when normalized to their predrug measures.

### Baclofen inhibited cardiovascular responses to UAB

**Fig. 2:**
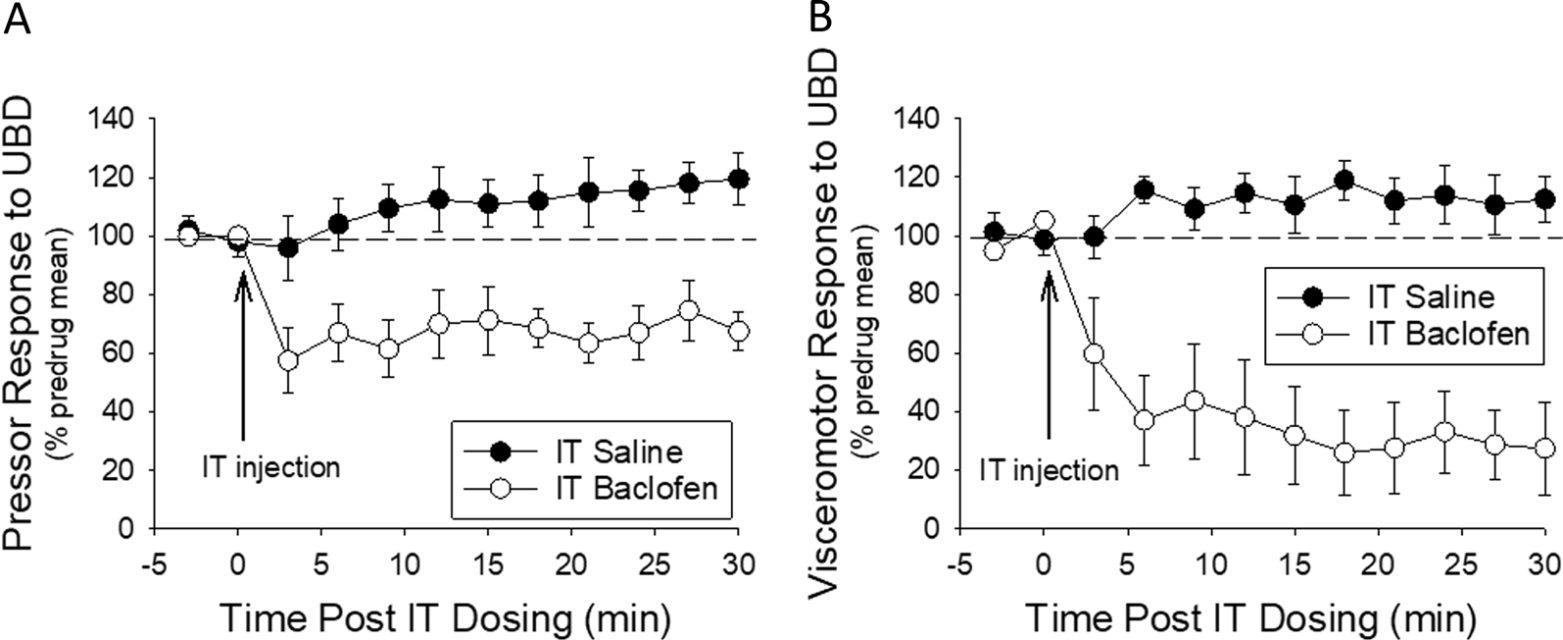
Time course of IT baclofen effect on reflex responses to UBD. Graphs depict the rapid onset and duration of inhibitory effect of a single 40 ng dose of IT baclofen or IT normal saline on repeated pressor (**A**) and viscero-motor (**B**) responses evoked by a urinary bladder distension (60 mm Hg, 20 s, every 3 min) normalized as percentage of pre-drug responses. N = 6/group

UBD (60 mm Hg, 20 s) produced a reliable pressor response that averaged 22.5 ± 1.3 mm Hg in the 12 rats studied. Figure 2A shows the analgesic effect of 40 ng of IT baclofen on pressor responses evoked by a 60 mmHg UBD stimulus presented every 3 min when compared with IT saline-treated controls. In these same rats the mean VMR also significantly decreased as a function of time after baclofen administration (Fig. 2B).

### Blockade by the receptor antagonist CGP 35348

Table 2 reports the effects of sequential IT administration of saline or 30 µg of the GABA_B_ receptor antagonist CGP35348 followed by the IT administration of 40 ng of baclofen or saline in the relevant comparison groups. In Sequence 1—40 ng of baclofen produced significant suppression of the AUC response to UBD when it followed an IT saline administration. In Sequence 2—IT CGP35348 significantly attenuated the suppressive action of baclofen. However in Sequence 3, when administered by itself, CGP35348 did not produce significant effects. Likewise in Sequence 4—repeated measures of the VMRs were unaffected when only saline was administered IT. There was no significant quantitative or qualitative difference in the effects of the different drugs on different rat bladder inflammation pretreatment groups (i.e., NBI Plus ABI Group vs. No NBI Plus ABI Group vs. No NBI-No ABI Group).

**Table 2:** Effect of IT baclofen and a GABA_B_ receptor antagonist on responses to urinary bladder distension in rats with and without previous episode of bladder inflammation as neonates and/or as adults (data presented as % baseline AUC)

Pretreatments	NBI	No NBI	No NBI
	Plus ABI	Plus ABI	No ABI
*SEQUENCE 1*			
Saline IT	145.8 ± 24.0	110.1 ± 5.7	132.1 ± 15.9
Then 40 ng IT baclofen	46.1 ± 10.9*	63.5 ± 12.8*	64.7 ± 17.3*
*SEQUENCE 2*			
CGP35348 IT (30 mcg)	104.1 ± 12.7	101.2 ± 6.0	138.5 ± 25.0
Then 40 ng IT baclofen	90.7 ± 11.3^#^	102.1 ± 5.7^#^	134.8 ± 28.5^#^
*SEQUENCE 3*			
CGP35348 IT (30 mcg)	106.2 ± 13.8	130.7 ± 12.7	123.8 ± 13.5
Then saline IT	112.7 ± 19.7	144.1 ± 12.1	129.4 ± 27.4
*SEQUENCE 4*			
Saline IT	110.8 ± 9.3	111.2 ± 10.2	129.4 ± 16.3
Then Saline IT	88.3 ± 18.2	94.9 ± 9.9	143.3 ± 19.4

Data represents mean ± SEM

CPG35348 is a selective GABA_B_ receptor antagonist

*NBI* neonatal bladder inflammation—pretreatment as defined in section “Neonatal bladder inflammation (NBI)” of text; *ABI* adult bladder inflammation—pretreatment as defined in section “Adult bladder inflammation (ABI)” of text; *mcg* microgram, *ng* nanogram

*Statistically significant change (*p* < 0.05, paired t-test) from previous measure. N = 8–9/group

^#^Statistically significant difference from Saline-IT Baclofen treated rats in same Pretreatment category (*p* < 0.05, unpaired t-test)

### IT baclofen still effective after chronic infusion

Baclofen or saline were infused continuously for 7 days via implanted osmotic minipumps to evaluate whether tolerance to the effects of baclofen would be apparent. As noted in Table 3, the dose-dependent inhibition of VMRs by cumulative doses of IT baclofen in rats which had previously received 7 day infusions of IT baclofen, when compared with similar measures in those rats which had received a 7 day infusion of IT saline demonstrated no statistically significant quantitative or qualitative differences.

**Table 3:** Dose-dependent effects of cumulative doses of baclofen on visceromotor responses to urinary bladder distension measured as % baseline AUC in rats which received 7 day IT baclofen (20 ng/h) or IT saline infusions prior to testing

Cumulative dose IT	Pretreament	
	7 day infusion of baclofen IT (n = 7)	7 day infusion of saline IT (n = 6)
Baclofen 10 ng	112.5 ± 19.4	102.1 ± 13.9
Baclofen 20 ng	115.5 ± 20.3	83.6 ± 14.7
Baclofen 40 ng	57.4 ± 17.2	53.8 ± 15.5
Baclofen 80 ng	28.3 ± 9.5	17.5 ± 8.1
Baclofen 160 ng	11.2 ± 5.6	2.2 ± 0.8

Data represents mean ± SEM. AUC indicates Area-Under-the-Curve, a statistic describing vigor of visceromotor responses to graded urinary bladder distension (10–60 mm Hg, 20 s). See text for more complete description. There were no statistically significant differences between Baclofen-Infused and Saline-Infused rats. All rats had received Neonatal Bladder Inflammation and Adult Bladder Inflammation pretreatments

### IT baclofen inhibits UBD-evoked activity of spinal dorsal horn neurons

**Fig. 3:**
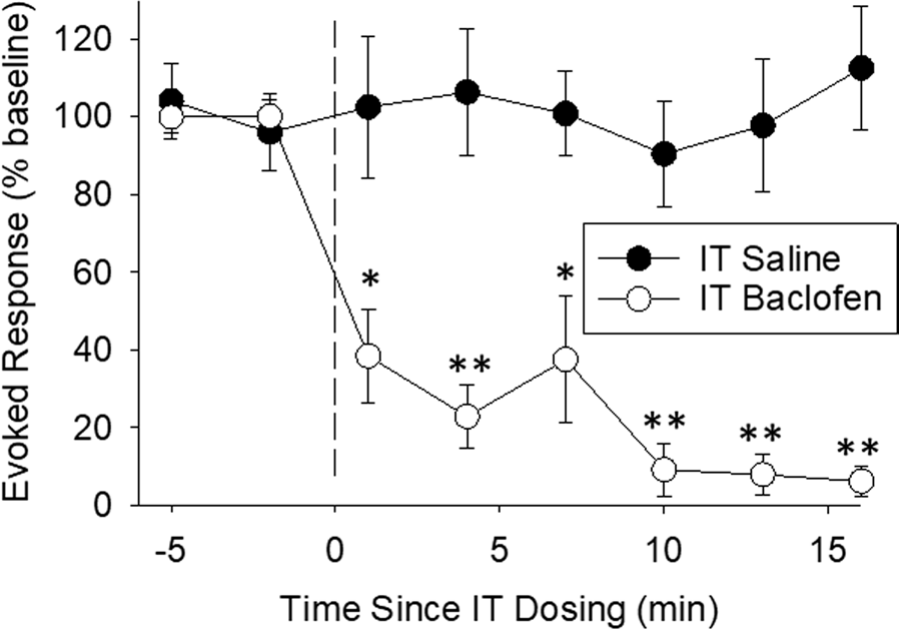
Time course of IT baclofen effect on spinal dorsal horn neuronal responses to UBD. Graph depict the rapid onset and duration of a single 160 ng dose of IT baclofen or IT normal saline applied topically to the dorsal surface of the spinal cord on repeated neuronal responses to urinary bladder distension (60 mm Hg, 20 s, every 3 min) normalized as percentage of pre-drug responses. See text for more complete description. N = 10/group. *, **p < 0.05 and *p* < 0.01 respectively for post hoc comparisons with IT saline-treated rats

As evidenced by Fig. 3, a single 160 ng dose of baclofen applied topically to the dorsal surface of the exposed spinal cord produced consistent, statistically significant inhibition of neuronal responses evoked by UBD when compared with responses following topical saline administration. Effects on Spontaneous Activity were more variable; changes in Spontaneous Activity due to baclofen administration failed to reach statistical significance. The neurons studied were located 0.515 ± 0.09 mm deep to the cord dorsum: all had convergent cutaneous receptive fields demonstrating either excitation by both noxious and non-noxious cutaneous stimuli (Class 2) or excitation only by noxious stimuli (Class 3). Specifically, the Baclofen-treated Group consisted of eight Class 2 and two Class 3 neurons and the Saline-treated Group consisted of seven Class 2 and three Class 3 neurons. The two treatment groups also consisted of neurons that were equally distributed according to previous classifications of UBD-excited neurons which used heterosegmental noxious stimuli as a discriminating factor [39]. In that classification scheme, Type I neurons are those which are inhibited by heterosegmental noxious stimuli whereas Type II neurons are neurons which are not inhibited by such stimuli. Using this classification scheme, both the Baclofen-treated Group and the Saline-treated Group consisted of five Type I and five Type II neurons. Baclofen had similar inhibitory effects on both the Type I and Type II neuronal subgroups with no statistically significant differences noted. None of the rats studied had experienced bladder inflammation. A more complete characterization of spinal neurons responsive to UBD has been presented elsewhere [39].

## Discussion

The most important finding of the present study was that the non-opioid agent, baclofen, inhibited responses to UBD in a variety of models of bladder nociception. These actions included spinal sites of action and the activation of GABA_B_ receptors. To the best of our knowledge, this is the first report of such a finding in bladder nociceptive systems. We studied these effects in multiple models (e.g., reflex responses, neuronal responses, inflammatory and noninflammatory conditions) of bladder nociception and observed similar effects in all models suggesting this pharmacology may be generalized to many different etiologies of bladder pain and is not just a model-specific antihyperalgesic therapy. Given its long history of clinical use and low toxicity, a trial in humans for the treatment of bladder pain would seem an appropriate next step [41].

The models studied here included one in which rats experience neonatal bladder inflammation and then receive a second bladder inflammatory challenge as adults [37]. It is thought that this model may be particularly relevant to the disorder interstitial cystitis/bladder pain syndrome [34] in that it is associated with multiple features of IC/BPS including the presence of increased micturition rates, a functionally small capacity hypersensitive bladder, altered bladder neurochemistry, the presence of vascular fragility of submuscosal tissues following prolonged hydrodistension, the presence of increased pelvic floor muscular tone, increased responsiveness to acute stress and increased responsiveness to intravesical cold and potassium-containing fluids [37, 42]. One would think that the pelvic floor spasm noted in these patient populations should be justification enough (as an antispastic agent) for the clinical assessment of baclofen. Indeed, targeting pelvic floor hypertonicity associated with chronic pelvic pain syndromes has been the strategy of mainstay therapies such as myofascial physical therapy [46–49] and new clinical research involving local injections of botulinum toxin with and without physical therapy [50–52].

Effects of baclofen on urodynamic measures that are independent of pain has been extensively investigated in non-humans [53–61] and humans [60, 61] most commonly in the context of neurological injury [62–71] or for symptoms of overactive bladder [59, 60, 72]. Inhibitory effects of baclofen, administered either systemically or spinally, on urodynamic measures was the consistent observation. Given that all dorsal horn neurons excited by UBD were robustly inhibited, this result should not be surprising as these neurons are likely important in both nociceptive and non-nociceptive bladder sensory functions. Despite all of the above listed studies, the present report is the first to specifically use models of bladder nociception in the characterization of baclofen effects and therefore establish a relevance to clinical pain.

As noted previously, baclofen has been demonstrated to have significant benefits in association with reduction of addictive behaviors. The presumed mechanisms for its actions on addictive behaviors is an interaction with dopaminergic neurons of the ventral tegmental area [77] with a subsequent reduction in dopamine release in the nucleus accumbens. As such, it is notable that Fadda et al [78] observed that baclofen blocked morphine-induced dopamine release at the nucleus accumbens. Baclofen also reduced nicotine- and morphine self-administration in rats [79] whereas a GABA_B_ receptor antagonist increased morphine administration [80]. Baclofen administered into the locus coeruleus attenuated morphine withdrawal signs [81] and in a randomized, double-blind placebo-controlled clinical trial Assadi et al [82] used baclofen for the maintenance treatment of opioid dependence and found baclofen to be superior over placebo in terms of opiate withdrawal syndrome and depressive symptoms. In that study, trends towards reductions in opioid craving and self-reported opioid and alcohol use were also noted but not proven. Given the clinical literature demonstrating that baclofen may potentiate opioid antinociceptive effects [25, 26] one can imagine co-administration of baclofen with opioid analgesics [83] so that one might optimize analgesia and minimize reward system activation. DeFeudis [84] suggested that GABAergic drugs might have a role in both analgesia and drug addictions, particularly related to opioids but did not go so far as to suggest co-administration. Future studies would be needed to test this supposition. Studies in opioid-tolerant rats could also assess whether there could be particular clinical benefits in subjects already treated with opioids prior to a trial of baclofen.

In determining the potential analgesic benefit of baclofen for human use, it is important to consider the most efficacious route of administration that produces strong analgesia with the fewest side effects or toxicities. For example, odd reactions to systemic baclofen include things such as the induction of hiccup-like respirations [85] or diabetes insipidus [86]. In rats, intraventricular baclofen impaired memory [87]. Toxicity has been reported in some studies with CNS effects (loss of consciousness, delirium, hypertension) but these have been predominantly related to withdrawal [88] or excessive dosing (e.g. [89]). Oral baclofen may be dose-limited by side effects (e.g. drowsiness, dizziness, or weakness) when treating patients with spasticity. When administered by IT infusion baclofen has been demonstrated to be an effective therapy for severe spasticity associated with multiple sclerosis, spinal cord injury, cerebral palsy and traumatic/ischemic brain injury [90–92]. As baclofen is zwitterionic at physiologic pH, by bypassing the blood–brain barrier and directly targeting the central nervous system, when administered IT, efficacy can be maximized and side effects can be minimized significantly in many patients. For this reason, the present study examined the effects of the IT administration of baclofen. A comparison of the potency of baclofen needed to inhibit UBD-evoked responses demonstrated that IT dosing was approximately 20,000 times as potent as IP administration. This suggests that the most beneficial route of administration in humans may be spinal delivery because it is primarily restricted to the spinal segment to which it is administered and could potentially avoid issues related to CNS effects and toxicity due to high dosing. One option for a proof-of-concept clinical trial would be to administer a single IT dose of baclofen (versus placebo) in a fashion similar to what is described in the product insert for the drug [92].

It is clear that baclofen produces spinal antinociception given its inhibitory effects on spinal dorsal horn neurons as well as the fact that the IT baclofen inhibited cardiovascular responses evoked by UBD. That said, a limitation of the present study is that the main endpoints in most of the study were VMRs, a motor reflex. As a consequence, it must be considered whether baclofen, administered spinally, may have also been acting on motoneurons thereby increasing its potency in that assay. Spinal administration would also likely miss some of the potential behavioral benefits (e.g. addiction prevention) associated with systemic baclofen use. As in all analgesic regimens, particularly opioids, one must be concerned with the development of tolerance to the administered agent. It is therefore fortunate that tolerance did not appear to be a problem after a seven day continuous infusion of “chronic” IT baclofen prior to assessing the potency and efficacy of acutely administered IT baclofen. Longer infusions would be necessary to fully assess this, but these results are consistent with the clinical observations by others [93, 94] that tolerance to the continuous infusion of baclofen does not appear to be a problem.

The precise mechanisms of baclofen’s antinociceptive actions are not known. The present study gave evidence that it is through spinal GABA_B_ receptor activation since the IT preadministration of the antagonist CGP36348 blocked subsequent IT baclofen effects, but the precise sites of action are not well defined. There is some suggestion in the literature that GABA_B_ receptors may interact with spinal Substance P and its receptors [95, 96] but numerous other neurotransmitters have also been implicated. Baclofen’s analgesic effects may be acting via the same mechanism by which segmental and heterosegmenal noxious stimuli produce inhibition (e.g. [97]), a phenomenon referred to as nocigenic inhibition [98] or Noxious Stimulus Induced Analgesia, since such inhibition has been demonstrated to act via GABA_B_ and mu opioid mechanisms [17]. In the spinal substantia gelatinosa activation of GABA_B_ receptors presynaptically block neurotransmitter release [99] and NK1 receptor expression [96]. Other supraspinal sites of action related to pain include the rostral agranular insular cortex [12] and lateral preoptic area [21]. Functional magnetic resonance imaging using continuous arterial spin labelling of the brain in humans before and after 21 days of systemic baclofen treatment (20 p.o. QID) demonstrated reduced regional cerebral bloodflow (rCBF) in the ventral striatum and medial prefrontal cortex and increased rCBF in the lateral orbital frontal cortex, (a region involved in suppressing previously rewarded behavior) and cerebellum. rCBF was also blunted in the insula bilaterally, a site commonly activated in painful conditions [100]. The supraspinal analgesic effects of baclofen appear to act via adrenergic and opioidergic spinal mechanisms [12, 87, 101] with potentially a cholinergic and GABA_A_ receptor contribution [16, 20]. Intrathecal nociceptin antagonized baclofen-induced analgesia in a mouse tail flick assay [10] and baclofen-induced analgesia was absent in GIRK2 knockout mice [102] all suggesting the potential for a complex pharmacology.

## Conclusions

In summary, the compound baclofen, a drug currently approved for clinical use, which can be administered systemically or spinally, was demonstrated to have antinociceptive effects in animal models of bladder pain. This supports the assertion that a clinical trial for the treatment of bladder pain should be undertaken.

## Data Availability

Data used and reported in the present study are available upon reasonable written request to T. J. Ness, UAB Department of Anesthesiology, BMR2-208, 901 19th St. S. Birmingham, AL 35222.

## Abbreviations

ABI: Adult bladder inflammation
ANOVA: Analysis of variance
AUC: Area Under the Curve
BPS: Bladder pain syndrome
CNS: Central nervous system
CGP35348: A GABA_B_ receptor antagonist
GABA: Gamma amino butyric acid
gms: Grams
h: Hour
IC: Interstitial cystitis
IP: Intraperitoneal
IT: Intrathecal
ITI: Inter-trial interval
kg: Kilogram
L6-S6: Lumbar level six through sacral level two
min: Minutes
mg: Milligram
mm: Millimeter
MOhm: Mega ohm
NBI: Neonatal bladder inflammation
ng: Nanogram
P14-16: Postnatal days 14–16
PE: Polyethylene
p.o.: By mouth
QID: Four times per day
rCBF: Regional cerebral bloodflow
s: Seconds
s.c.: Subcutaneous
SEM: Standard error of the mean
UBD: Urinary bladder distension
VMR: Visceromotor reflex
µg: Microgram
µl: Microliter
